# MyoD-induced reprogramming of human fibroblasts and urinary stem cells *in vitro*: protocols and their applications

**DOI:** 10.3389/fphys.2023.1145047

**Published:** 2023-05-17

**Authors:** Rachele Rossi, Silvia Torelli, Pierpaolo Ala, William Weston, Jennifer Morgan, Jyoti Malhotra, Francesco Muntoni

**Affiliations:** ^1^ The Dubowitz Neuromuscular Centre, UCL Great Ormond Street Institute of Child Health, London, United Kingdom; ^2^ National Institute for Health Research, Great Ormond Street Institute of Child Health Biomedical Research Centre, University College London, London, United Kingdom; ^3^ Sarepta Therapeutics Inc., Cambridge, MA, United States

**Keywords:** duchenne muscular dystrophy, MyoD-induction, reprogramming, fibroblasts, urinary stem cells, lentivirus

## Abstract

The conversion of fibroblasts into myogenic cells is a powerful tool to both develop and test therapeutic strategies and to perform in-depth investigations of neuromuscular disorders, avoiding the need for muscle biopsies. We developed an easy, reproducible, and high-efficiency lentivirus-mediated transdifferentiation protocol, that can be used to convert healthy donor fibroblasts and a promising new cellular model, urinary stem cells (USCs), into myoblasts, that can be further differentiated into multinucleated myotubes *in vitro*. Transcriptome and proteome profiling of specific muscle markers (desmin, myosin, dystrophin) was performed to characterize both the myoblasts and myotubes derived from each cell type and to test the transdifferentiation-inducing capacity of MYOD1 in fibroblasts and USCs. Specifically, the Duchenne muscular dystrophy (DMD) transcripts and proteins, including both the full-length Dp427 and the short Dp71 isoform, were evaluated. The protocol was firstly developed in healthy donor fibroblasts and USCs and then used to convert DMD patients’ fibroblasts, with the aim of testing the efficacy of an antisense drug *in vitro*. Technical issues, limitations, and problems are explained and discussed. We demonstrate that MyoD-induced-fibroblasts and USCs are a useful *in vitro* model of myogenic cells to investigate possible therapies for neuromuscular diseases.

## 1 Introduction

Duchenne muscular dystrophy (DMD) (OMIM # 310200) is a rare X-linked genetic disease affecting 21.4 children per 100,000 live male births. It is caused by mutations affecting the *DMD* gene resulting in reduction or complete absence of the related protein: dystrophin (DYS) (Crisafulli et al., 2020) (Muntoni et al., 2003). The most common mutations in *DMD* are large deletions and duplications, followed by small ins/del, point mutations, and splicing or deep intronic copy number variations (CNVs)/small mutations. Although the most frequent mutations are easily identifiable by a DNA test (i.e., MLPA, Sanger sequencing, gene panel), approximately 1% of atypical mutations require RNA analysis to be identified (Neri et al., 2020). In recent years, many therapeutic strategies have been developed aiming to restore functional dystrophin in DMD muscles or to ameliorate the DMD patient’s symptoms (Iftikhar et al., 2021). Other therapeutic strategies are now in development or in clinical trials (Fortunato et al., 2021), requiring a robust and reliable *in-vitro* model for their development. Neuromuscular disease-specific *in-vitro* models are more readily available than *in vivo* models, and are relevant for mutations present in patients that do not occur in existing rodent models (Fralish et al., 2021). However, these cellular models are frequently obtained from myoblasts isolated from skeletal muscle biopsies (San Miguel and Vargas, 2006). Muscle biopsies are also required to perform the in-depth analysis on RNA to study and confirm the occurrence of atypical mutations. Unfortunately, muscle biopsies are invasive procedures, and require particular attention in boys affected by DMD in view of the well-known general anesthesia related adverse events in this population (Thavorntanaburt et al., 2018). In this scenario, cellular genetic reprogramming offers a great advantage, allowing the use of other mesoderm derived cells instead of myoblasts derived from skeletal muscle biopsies. Indeed, the *MyoD* gene, the master regulatory factor, is able, once delivered into non-muscle lineage cells, such as dermal fibroblasts, to transdifferentiate them into muscle-like cells able to reproduce the disease phenotypic characteristics (Fujii et al., 2006). The most common delivery system is viral vector-mediated, but it has some limitations. For example, retroviral vectors have a very low transduction efficiency in slow-growing cells (Roest et al., 1999), whereas adenoviral vectors potentially offer a better transduction efficiency but exhibit cytotoxicity at high titres, require expression of the receptor of interest in the cells under study and do not integrate, leading to dilution following cell division (Fujii et al., 2006). To address these issues, we have developed a lentiviral vector, that is able to transduce both dividing and non-dividing cells, and efficiently and is reproducibly capable to reprogram dermal fibroblasts, derived from a skin biopsy, into myotubes *in vitro*. We also used the same lentiviral vector carrying the *MyoD* gene to transfect urinary stem cells (USCs) (Falzarano et al., 2016), to explore an entirely non-invasive cellular model. Aiming to test a DMD morpholino antisense oligomer (PMO) to skip *DMD* exon 53, golodirsen (Anwar and Yokota, 2020), we optimized an *in vitro* strategy of drug administration in MyoD-lentiviral transfected fibroblasts from both healthy donors and DMD patients. Protein and RNA extracted from these transfected and treated fibroblasts were analysed by qPCR with TaqMan systems and with highly efficient capillary Western blot (Wes), to evaluate the drug efficacy on DMD transcript and dystrophin protein restoration (Rossi et al., 2021) (Rossi et al., 2022). Considerations about the effect of transdifferentiation on DMD transcripts and DYS expression on healthy donor fibroblasts and USCs are discussed. We also characterized in detail two dystrophin protein isoforms, the short Dp71 and the full-length Dp427 m expression in these cellular models. Previous work had suggested that Dp71 expression is ubiquitously expressed with the exception of adult differentiated skeletal muscle, Dp427 m is the major isoform expressed in skeletal muscle fibers and it is only expressed in myofibers and muscle stem cells (de León et al., 2005). Thus, we analysed Dp71 and Dp427 m transcript and protein expression in induced and non-induced fibroblasts and urinary stem cells.

## 2 Materials and equipment

### 2.1 Materials, chemical

• DMEM high-glucose (Gibco, cat. no. 41966-052)
• Fetal bovine serum (FBS) (GIBCO) (Thermo Fisher, cat no. 26140)
• Horse serum (HS) (Thermo Fisher, cat no. 16050130)
• Penicillin/streptomycin (Pen/Strep) (Sigma, cat. no. P0781)
• Mega Cell (Sigma, cat. no. M3942)
• Non-Essential Amino Acids (NEAA) (Gibco, cat. no. 11140-050)
• 100X Glutamax (Gibco, cat. no. 35050-038)
• Skeletal Muscle Cell Differentiation Medium, ready to use, (Promocell, cat. no. C-23061)
• Trypsin-EDTA (Gibco, cat no. 15400-050)
• Phosphate-buffered saline (PBS) (Gibco, cat. no. 14190-094)
• Corning Matrigel Basement Membrane Matrix Growth Factor Reduced (Corning, cat. no. 354230)
• Doxycycline Hyclate (Sigma-Aldrich, cat. no. D9891)
• Puromycin: (Gibco, cat. no. A11138-03)
• Recombinant human epidermal growth factor (hEGF) (Lonza Bioscience, cat. no. CC-4107)
• Basic recombinant human fibroblast growth factor (bFGF) (Prospec-Tany, cat. no. CYT-218)
• Platelet-derived growth factor (PDGF-AB) (Prospec-Tany, cat. no. CYT-342)
• REBM Basal Medium (Lonza, cat. no.CC-3191)
• REGM renal epithelial SingleQuots Kit (Lonza, cat. no.CC-4127)
• Gibco Ham’s F-12 Nutrient Mix (Gibco, cat. no. 11765054)
• Antibiotic/antimycotic solution (Sigma-Aldrich, cat. no. 120M0827)
• Ultrapure water with 0.1% gelatin (gelatin) (Millipore, cat. no. ECM0011B)
• Complete Protease Inhibitor Cocktail (Roche, cat. no. 11697498001)
• Plasmid: LV-TRE-VP64 mouse MyoD-T2A-dsRedExpress2 (donated by Dr Charles Gersbach but commercially available from Addgene plasmid no. 60625)
• Antisense PMO, Stock solution 1 mM.
• Endo-porter (PEG, Gene-Tools, cat. no. 2922498000)
• EndoFree Plasmid Giga Kit (QUIGEN, cat. no. 12391)
• RNeasy Mini Kit (QUIAGEN, cat. no. 74104)
• DNase I enzyme, Deoxyribonuclease I (Thermo fisher, cat. no.18068015)
• High-Capacity cDNA Reverse Transcription Kit (Applied Biosystems, cat. no. 4368814)
• TaqMan Universal PCR Master Mix (Thermo Fischer scientific, cat. no 4304437)
• Customized TaqMan systems (Thermo Fischer scientific, cat. no.4331348)
• Commercial TaqMan system for ACTBL2 gene, Hs01101944_s1 (Thermo Fischer scientific, cat. no.4331182)
• DNAse enzyme (Thermo Fisher cat. no. 1847019)
• Pierce BCA kit (Thermo Fisher Scientific, cat. no. 23250)
• Rabbit anti-dystrophin antibody (ab15277, Abcam, UK, dilution 1/50; ab154168)
• Anti Mf20 antibody (Developmental Studies Hybridoma Bank, United States, 1:200)
• Anti Desmin antibody clone D33 (Dako, Agilent, 1:50)
• Anti-Human Fibroblast Surface Protein (Sigma, 1:500)
• Anti-rabbit (DM-001) and anti-mouse (DM-002) detection modules (Protein Simple, Bio-Techne)
• 66-440kD WES separation module 8 × 25 capillary cartridge (SM-W008, Bio-Techne)
• EZ Standard Pack 3 (PS-ST03EZ-8, Bio-techne)
• Alexa Fluor-488 goat anti mouse and anti rabbit antibodies (1:1000, ThermoFisher, United States)

### 2.2 Materials, single-use plastic

• Steripipettes
• Counting Slides
• 15 mL sterile Falcon tubes
• 50 mL sterilie Falcon tubes
• T75 sterile flasks
• T175 sterile flasks
• 12 sterile well plate, flat bottom
• 6 sterile well plate, flat bottom

### 2.3 Equipment

• Wes system (ProteinSimple, Bio-Techne, United States)
• Real-Time PCR System (StepOnePlus™, Applied Biosystems, MA, United States)

### 2.4 Reagent setup

• Fibroblast Growth Medium: DMEM high-glucose, 10% FBS, 1% Pen/Strep
• Priming Differentiation Medium (MegaCell): Mega Cell, 2% FBS, 1% NEAA, 1% 100X Glutamax
• Secondary Differentiation Medium (PromoCell): PromoCell Skeletal Muscle Cell Differentiation Medium added with the provided supplement.
• Mesenchymal Proliferation Medium: DMEM high glucose, 10% FBS, 1% 100X GlutaMAX, 1% NEAA, 1% antibiotic/antimycotic solution, 5 ng/mL bFGF, 5 ng/mL PDGF-AB, 5 ng/mL EGF.
• USC Proliferation Medium: RE cell basal medium added with Renal Epithelial SingleQuots Kit (following company instruction) and Mesenchymal proliferation medium mixed at a 1:1 ratio.
• USC transfection Medium: DMEM high-glucose, 2% HS, 1% antibiotic/antimycotic solution.
• Corning Matrigel Basement Membrane working stock (Matrigel): to dilute the master stock to 0.1 mg/mL in DMEM-serum free, keeping Matrigel master stock on ice, see detailed protocol (Muses et al., 2011). Working solution should be stored at 4°C for no longer than 1 month.
• Puromycin working stock (Puromycin): experimentally determined (see below) to be 0.75 μg/mL with a maximum ratio of 10,000cells/100 μL medium.
• Doxycycline Hyclate working stock (Doxycycline): 3 μg/mL stock solution diluted to 10^3 diluted (1 μL in 10 mL of medium).
• Lysis buffer A: urea 4M, Tris 125 mM pH 6.8, SDS 4%.

## 3 Methods

### 3.1 Cells

This work was performed under the NHS National Research Ethics: setting up of a rare diseases biological samples bank (biobank) for research to facilitate pharmacological, gene and cell therapy trials in neuromuscular disorders (REC reference number: 06/Q0406/33), and the use of cells as a model system to study pathogenesis and therapeutic strategies for Neuromuscular Disorders (REC reference 13/LO/1826).

1 HEK-293-T Cell Line (Merck KGaA, Darmstadt, Germany, cat.no. 12022001-1VL) and 1 Lenti-X™ 293T Cell Line (Takara, 2022, cat.no. 632180) were used for large-scale vector production (Albrecht et al., 2015.). 1 Human fibrosarcoma HT1080 cell line (ATCC, cat. no. CCL-121) was used to perform lentiviral titration and to establish the protocol. Fibroblasts from 5 human adult healthy donors, were isolated from skin biopsies collected after obtaining written informed consent, to test the protocol reproducibility and to perform the RNA and protein tests. Urinary stem cells were previously isolated from 2 human adult healthy donors and cultured following the Falzarano et al. protocol (Falzarano et al., 2016).

### 3.2 Lentivirus and protocol set up

#### 3.2.1 Plasmid

In this study was used the plasmid LV-TRE-VP64 mouse MyoD-T2A-dsRedExpress2 kindly donated by Dr Charles Gersbach. Briefly we cloned the MyoD gene fused with Vp64 activation domain into a Tet-ON lentiviral vector. The lentiviral vector constitutively expresses the reverse tetracycline transactivator (rtTA2S-M2) and the puromycin resistance gene (PuroR) from the human phosphoglycerate kinase (hPGK) promoter. It also contains a DsRed Cassette downstream of the tetracycline response element (TRE) promoter. The rtTA2S-M2 binds to the TRE and activates expression of the downstream genes in the presence of doxycycline. Vp64 enhances the expression of the downstream gene, the MyoD factor. The puromycin selection cassette enables enrichment of transduced cells, while the DsRed cassette enables assessment of transduction efficiency.

#### 3.2.2 Lentiviral production

Lentiviral production was undertaken using either HEK293T or the commercial LentiX HEK293T cell lines. A 24-flask production system was utilized, with separate harvests and ultracentrifugation steps at 48 and 72 h. The time of lentiviral harvest is critical to the titer that will be achieved (TAKARA BIO INC.). The LentiX cell line was used in an attempt to increase viral titer, but it appears to give a slightly lower titer than the HEK293T cells (data not shown). All the transduction experiments were conducted using only the HEK293T 48h samples.

#### 3.2.3 Lentiviral titration

50,000 HT1080 cells (Schwartz et al., 2013) were transduced with two different volumes of lentivirus (Table 1) and cultured for 7 days (virus was added to cell cultures on day 1 and cells were collected on day 7), to ensure lentiviral integration. DNA was harvested, diluted to 20 ng/μL, and assessed by qPCR using both custom TaqMan system for the LTR virus gene and commercial TaqMan system for human beta actin genes, alongside plasmid serial 7-points dilution ranging from 10E^10 to 10E^3. qPCR experiment conditions are described in Kutner, *et al* (2009) (Kutner et al., 2009).

**TABLE 1:** MyoD Lentiviral Titres obtained using two different commercial cell lines, HEK293T and LentiX HEK293T. Titres were obtained using cells harvested after 48 h of culturing.

Cell Line	Lentiviral Titre
HEK293T (48h harvest)	7.10E^6
LentiX HEK293T (48h harvest)	5.21E^6

#### 3.2.4 Determination of the optimal multiplicity of infection (MOI)

**FIGURE 1:**
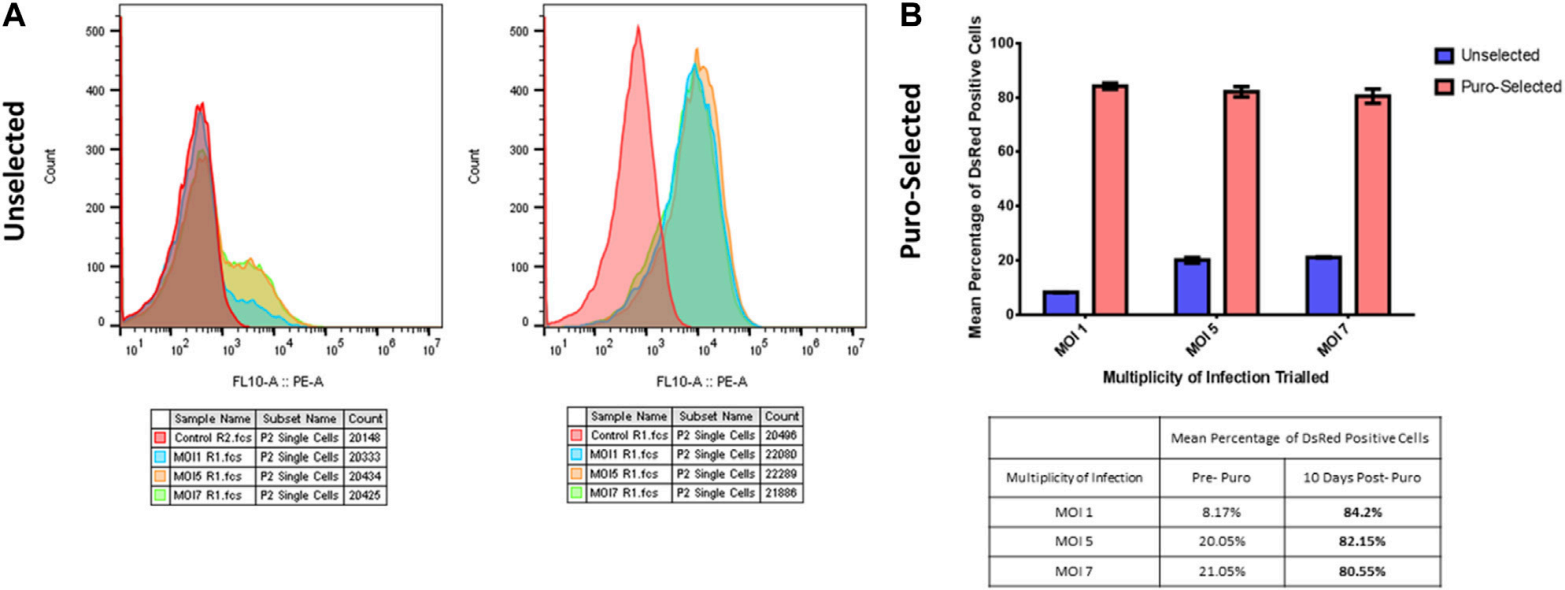
MOIs selection. **(A)** FACs profiles of human healthy fibroblasts infected with increasing multiplicity of infections (MOIs), pre (left graph) and post (right graph) puromycin selection. Red peak corresponds to non-transfected cells used as control sample, light blue peak to MOI 1, orange peak to MOI 5 and green peak to MOI 7. **(B)** DsRed positive cell count of human healthy fibroblasts infected with increasing multiplicity of infections (MOIs), pre (blue bars) and post (red bars) puromycin selection. Y-axis is the percentage of red signal from the DsRed Fluorescent label cassette present within the plasmid. The X-axis shows the different MOIs tested. Table below the graph shows the peak values.

Lentiviral transductions were undertaken on 1E^5 HT1080 cells, in 6 well-plates. To determine the baseline of transduction we analysed the DsRed reporter gene expression by FACS, using increasing multiplicity of infections (MOIs) (MOI 1, MOI 5, MOI 7) listed in Table 2. Each analysis was conducted pre and post 10 days of puromycin selection (Figure 1A). DsRed positive cells count take advantage of the DsRed fluorescent label cassette within plasmid #60625. After puromycin selection, MOI 1 and 5-transfected cells showed comparable DsRed expression as shown by FACs profiles and positive cell counts (Figures 1A, B). Cells transfected with MOI 7 had a slightly reduced number of cells compared to the other MOIs (Figure 1B). The DsRed positive cell count reveals a correlation between MOIs and the initial baseline transduction efficiency. However, following approximately 10 days of puromycin selection, the proportion of DsRed positive cells appeared to be similar between groups, especially between MOI 1 and 5. MOI 1 was selected to avoid cell stress and save virus stocks, achieving the same result compared to the other MOIs.

**TABLE 2:** Multiplicity Of Infection (MOI) volumes to optimize transduction with increasing MOIs.

HEK293T 48h (1E^5 cells)	Volumes
MOI 1	14.08ul
MOI 5	70.42ul
MOI 7	98.59ul

#### 3.2.5 Determination of the optimal puromycin concentration

The puromycin working concentration recommended by the manufacturer (Gibco) ranges from 0.2 to 5.0 μg/mL, although the company website reports toxicity in eukaryotic cells at concentrations of 1 μg/mL. We tested two concentrations: 1 μg/mL and 0.75 μg/mL. We found that a dose of 1 μg/mL results in reduced proliferation, granulations and markers of stress in transfected fibroblast populations. On the contrary, a dose of 0.75 μg/mL allows selection of transfected cells without interfering with proliferation or causing excessive cell stress. Thus, 0.75 μg/mL was chosen as working concentration for human fibroblast selection. Moreover, at this concentration, we found that puromycin causes toxicity if it is administrated with a ratio greater than 100 µL of medium containing 0.75 μg/mL of puromycin for 10,000 cells.

#### 3.2.6 Medium selection

**FIGURE 2:**
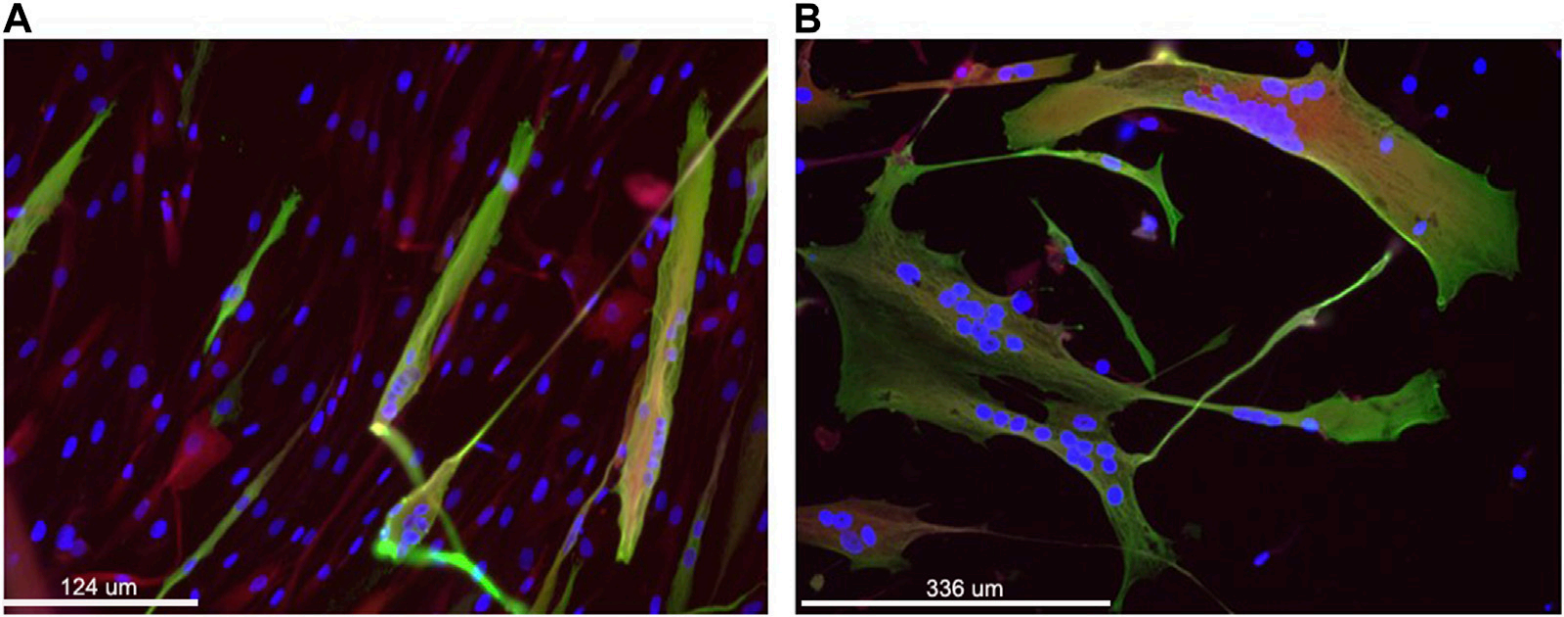
Immunofluorescence staining of transduced fibroblasts to assess MyoD-mediated differentiation and fusion of cells. Nuclei are stained in blue (DAPI), Sarcomeric myosin heavy chain in green using MF20 antibody and in red the residual DsRed associated with the transgene expression. Panel **(A)** shows cells grown for 10 days in primary culture medium and panel **(B)** shows cells grown for 10 days in the secondary culture medium. Magnification: bar 124 and 336 microns in panel A, B, respectively.

After puromycin selection (11 days in total), fibroblasts were seeded on Matrigel-coated flasks and differentiated using two different media (MegaCell and PromoCell differentiation medium), both containing doxycycline (0.1 μL/mL), to test their effect on myogenic differentiation. After 10 days, cells cultured with both media were analyzed for the expression of myosin as a myotube marker, by IHC (Figure 2). The analysis revealed that MegaCell medium resulted in better viability (Figure 2A), whereas PromoCell differentiation medium resulted in better fusion (Figure 2B). It was decided to move to a culture system using both media: MegaCell for 5 or 9 days (see below) from the start of transdifferentiation and PromoCell for the 5 remining days, for a total of 14 days in differentiation media, in order to achieve a reasonable viability and fusion.

### 3.3 Fibroblast MyoD transduction protocol

Expand human fibroblast cells to reach 70%–80% confluence, in a T75 flask containing 5 mL of fibroblast growth medium. Then, trypsinise cells (remove all medium, wash cells with 5 mL of PBS, add 3 mL of trypsin-EDTA to each flask, place in an incubator until the cells have detached (approximately 2- 3 min), inactivate trypsin with 5 mL of fresh growth medium), count and seed 10^^5^ cells/well in a 6 well plate, using 1mL/well of fibroblast growth medium. Gently agitate thawed lentiviral aliquots and pipette a volume consistent with the selected MOI. In this case we used 14.08 µL of MyoD Lentiviral prep “HEK293T 48hrs” at multiplicity of infection 1 (MOI 1) to infect 100,000 cells in 1 mL. Apply gently using a dropwise circular motion. Wet tips with medium before taking up the virus to reduce the possibility of the virus adhering to the plastic tip.

Place cells with freshly applied lentivirus into an incubator at (37°C/5% CO_2_) for 24 h. Ensure you close the incubator door gently. After 24 h of incubation, change the medium to 2 mL of fresh fibroblast growth medium and continue to grow the cells until they reach 80%–90% of confluency (around 3–4 days).

#### 3.3.1 Expansion of MyoD transduced fibroblasts from a 6 well plate

Trypsinise cells and plate them into a T75 flask with 10 mL of fresh fibroblast growth medium. Change the medium every 2–3 days. Examine cells every day, they should reach 70%–80% confluency in 3–4 days post seeding.

#### 3.3.2 Puromycin selection of MyoD transduced cells

Once cells have reached 70%–80% confluence, add puromycin (at previously determined concentration of 0.75 μg/mL) to fresh fibroblast growth medium. Change the medium with puromycin every 2–3 days, for either a total of 11 days, or until 80%–90% confluency is reached.

#### 3.3.3 MyoD trans-differentiation of transduced and cell selection

Coat wells of 6-well plates with Matrigel (add 700 µL of the Matrigel per well, rock the plate gently, put in an incubator 37°C/5% CO_2_ for 1 h, aspirate the excess Matrigel). Without allowing the Matrigel to dry out, seed 10^^5^ of puromycin-selected-cells per well in 2 mL of fibroblast growth medium without puromycin. Once cells are confluent, apply a Matrigel top layer to each well (aspirate all the medium, add 700 µL of Matrigel working solution to the cells, incubate for an hour, then remove excess Matrigel). From this step it is essential to work extremely carefully to prevent cell detachment. Add 2mL/well of Priming Differentiation Medium supplemented with doxycycline. This represents the start of the trans-differentiation process. Perform complete media changes using Priming Differentiation Medium supplemented with doxycycline every 2 -3 days. Myotube formation will be evident after 3–5 days. After 7 days from the start of the transdifferentiation (step 12), the top layer of Matrigel should be re-applied, as described in step 11. After 5 or 9 days* in Priming Differentiation medium, cells are transitioned to the Second Differentiation medium supplemented with doxycycline and maintained for 5 days * 9 days are recommended for functional tests such as drug tests or to evaluate proteins expressed at a later stage of myogenic differentiation.

### 3.4 USCs MyoD transduction protocol

Grow human USCs until they reach 80%–90% confluence, in a 6-well plate using 2 mL of USC proliferation medium/well. Trypsinise the cells (remove all medium, wash wells twice with 500 µL of trypsin-EDTA, add 500 µL of trypsin-EDTA to each well, place the plate in an incubator until the cells have detached (around 2- 3 min), inactivate trypsin with 2 ml of USC Proliferation medium/well). Mix cells from two wells in a 15 mL Falcon tube and count them. Centrifuge Falcon tubes (10 min at 400x g at room temperature (RT)), discard supernatant and wash pellets with 5 mL of USC transfection medium. Centrifuge Falcon tubes (10 min at 400x g, RT), discard supernatant and resuspend each pellet with 1 mL of USC transfection medium. Add 14.08 µL of MyoD Lentiviral preparation “HEK293T 48hrs” at MOI 1 to 10^^5^ cells. Perform the infection in Falcon tubes. Wet tips with medium before taking up the virus to reduce the possibility of the virus adhering to the plastic tip. Incubate Falcon tubes at 37°C/5% CO_2_ for an hour (lid should be not completely closed), mixing gently once, after 30 min of incubation. Coat T25 flasks with 0.1% gelatin (add 2.5 mL of gelatin/flask, rock the plate gently, put in an incubator at 37°C/5% CO_2_ for 30 min, aspirate the excess of gelatin, wash with 3 mL of PBS containing 1% of antibiotic/antimycotic solution, remove excess washing solution). Transfer infected cells from Falcon tubes into gelatin-coated T25 flasks (one flask for each Falcon tube). Place flasks into an incubator at (37°C/5% CO_2_) for 24 h. Ensure you close the incubator door gently. After 24 h of incubation, replace the medium with 2.5 mL of USC Proliferation medium and continue to grow cells until they reach 70%–80% confluency (about 2–3 days).

Follow Fibroblast MyoD transduction protocol from the “Puromycin Selection of MyoD Transduced Cells” section (step 7). For USCs is recommended not to exceed 7 passages.

### 3.5 Antisense oligonucleotide treatment of trans-differentiated fibroblasts

This drug test protocol is set up for an antisense phosphorodiamidate morpholino oligomers (PMO) inducing exon skipping in the *DMD* gene. Two doses of antisense were administered to cultured MyoD-induced fibroblasts, the first one for 24 h and the second for 48 h of incubation.

At day 6 from the start of trans-differentiation (step 17), remove medium from each well and apply 1mL/well of the Priming Differentiation medium supplemented with doxycycline. Apply 10µL/well of 1 mM PMO antisense using a circular dropwise motion.

Apply 6 µL of Endoporter using a circular dropwise motion, in every well. Incubate plates for 24 h then change the medium: 1mL/well of Priming Differentiation containing doxycycline. After 2 days, change medium to 2mL/well of the Second Differentiation medium supplemented with doxycycline. After 5 days (from the first antisense administration) remove medium from each well and replace it with 1mL/well of the Second Differentiation medium supplemented with doxycycline. Repeat steps 2 and 3. Incubate plates for 48 h (prolonged incubation time can be associated with toxicity). Cells are ready to be collected for further studies/analysis.

### 3.6 RNA extraction and analysis

RNAs were extracted from cells using the Quiagen RNeasy Micro Kit, treated with DNase I enzyme and retrotranscribed into cDNA by High-Capacity cDNA Reverse Transcription Kit. All the procedures were performed following the manufacturers’ instructions. cDNAs were analyzed using single system TaqMan qPCR, complementary to DMD Dp427 m 5′UTR exon 1 boundary (custom, sequence available under request), DMD Dp71 5′UTR exon 1 boundary (custom, sequence available under request). Different expression between DMD Dp427 m and Dp71 isoforms was evaluated using Ct, using the formula: 2^^-(Ct Dp427−Ct Dp71)^.

### 3.7 Capillary western immunoassay (Wes)

Proteins were extracted adding Lysis buffer A to the harvested cells (50 µL of Lysis buffer A/well), transferred to Eppendorf microtubes, boiled for 3 min, treated with DNAse enzyme (6µL/tube of DNAse incubated for 30 min at 4°C), centrifugation at 14,000 g for 10 min, at RT. Collect the supernatant and discard the pellet if you can see it. Protein quantification was performed using the Pierce BCA kit (ThermoFisher Scientific, United States). Capillary Western immunoassay (Wes) analysis was performed on a Wes system (ProteinSimple, Bio-Techne, United States) following the manufacturer’s instructions for the 66–440 kDa Separation Module, using 1 µg of sample lysate for each well. The following primary and secondary antibodies were used: rabbit anti-dystrophin antibody, anti MF20, anti-rabbit, and anti-mouse antibodies.

### 3.8 Immunostaining

Non-infected fibroblasts and differentiated MyoD lentiviral transfected fibroblasts and USCs were plated on coverslips coated with collagen. After 5 days in the Priming Differentiation medium and 5 days in the Secondary Differentiation medium (both doxycycline supplemented), cells were fixed in 4% paraformaldehyde for 10 min and then washed (3 × 10 min) in PBS. Coverslips were then incubated with primary antibodies against desmin, myosin heavy chain and dystrophin proteins for 1 h at RT and then washed (3 × 10 min) in PBS. After incubation with 488-conjugated secondary antibodies for 30 min at RT and washes in PBS, the coverslips were mounted in Hydromount + Dapi. Images were acquired using a Leica DMR microscope interfaced to MetaMorph (Molecular Devices).

## 4 Results

We demonstrated the reproducibility and efficacy of the MyoD-lentivirus protocol and used it to evaluate DMD Dp427 m and Dp71transcripts and proteins from healthy donor transduced fibroblasts and USCs.

### 4.1 MyoD-induced myogenic conversion of fibroblasts

**FIGURE 3:**
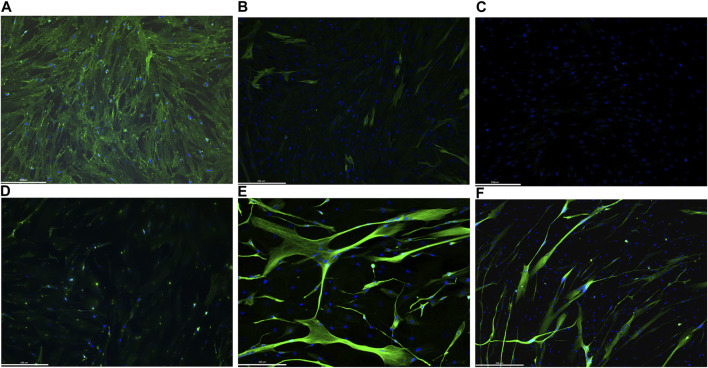
Representative images of immunofluorescence staining on 1 human healthy donor non-infected fibroblasts and differentiated MyoD lentivirally-transfected fibroblasts. Non-infected fibroblasts were immunolabelled with fibroblast surface protein antibodies **(A)**, desmin **(B)** and Mf20 **(C)**. Differentiated MyoD lentivirally-transfected fibroblasts were stained after 10 days in differentiation medium (5 days in priming and 5 days in secondary differentiation medium) with antibodies against fibroblast surface protein **(D)**, desmin **(E)** and Mf20 **(F)**. Nuclei are stained in blue (DAPI). Magnification: bar 336 microns.

In order to test the efficiency of our protocol in inducing myogenic conversion of fibroblasts, we immunostained (IHC) non-infected fibroblasts and differentiated MyoD lentiviral transfected fibroblasts for desmin and myosin heavy chain (MF20) expression. Desmin, an intermediate filament protein, is well-recognized to be one of the earliest expressed proteins during muscle development. Differentiated myotubes express high level of desmin, whereas myoblasts and satellite cells express lower levels of desmin, and fibroblasts do not express the protein (Paulin and Li., 2004). Sarcomeric myosin is expressed in striated and cardiac muscle where it confers contractile properties to the muscle fibers. Due to its tissue specificity, it has been used as a myoblast differentiation marker (Lee et al., 2019) (Shimizu et al., 1985). Immunostaining of non-infected fibroblasts, from one donor, shows only fibroblast surface protein expression (Figure 3A), while there was no signal for either desmin or myosin heavy chain (Figures 3B, C, respectively). On the contrary, after MyoD lentiviral infection and 10 days of transdifferentiation (5 days in primary differentiation medium and 5 days in secondary differentiation medium), fibroblasts express the two skeletal muscle markers, desmin and MF20 (Figures 3E, F, respectively). Moreover, immunostaining with an antibody against desmin (Figure 3E) reveals the characteristic elongate shape of myotubes formed from myoblast fusion. A slight background of fibroblast surface proteins expression was detected on differentiated MyoD lentiviral transfected fibroblasts (Figure 3D).

### 4.2 MyoD-induced myogenic conversion of USCs

**FIGURE 4:**
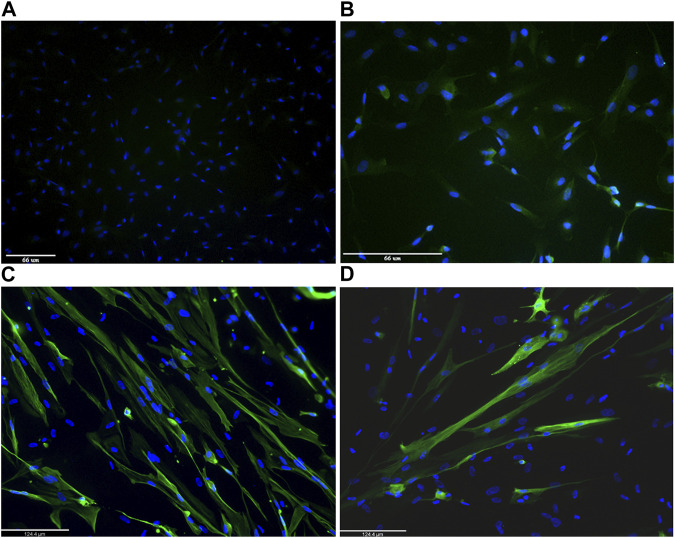
Representative images of immunofluorescence staining on 1 human healthy donor non-infected urinary stem cells (USCs) and differentiated MyoD lentivirally-transfected USCs. Non-infected USCs were immunolabelled with desmin **(A)** and Mf20 **(B)**. Differentiated MyoD lentivirally-transfected USCs were stained after 10 days in differentiation medium (5 days in Priming Differentiation Medium and 5 days in Secondary Differentiation Medium) with antibodies against desmin **(C)** and Mf20 **(D)**. Nuclei are stained in blue (DAPI). Magnification: bar 66 microns in panels A and B and bar 124.4 microns in panels C and D.

The two skeletal muscle markers, desmin and MF20, were also used to test the myogenic differentiation process on MyoD lentiviral infected and non-infected USC cultures. Figure 4 shows immunostaining of USCs from one healthy donor. Non-infected USCs do not express either desmin (Figure 4A) or MF20 (Figure 4B). Conversely, differentiated MyoD lentiviral transfected USCs, immunostained after 10 days in differentiation media (5 days in primary and 5 days in secondary differentiation medium) showed signal from both antibodies desmin (Figure 4C) and MF20 (Figure 4D).

### 4.3 Myosin heavy chain, sarcomere (MHC) capillary western blot (Wes)

**FIGURE 5:**
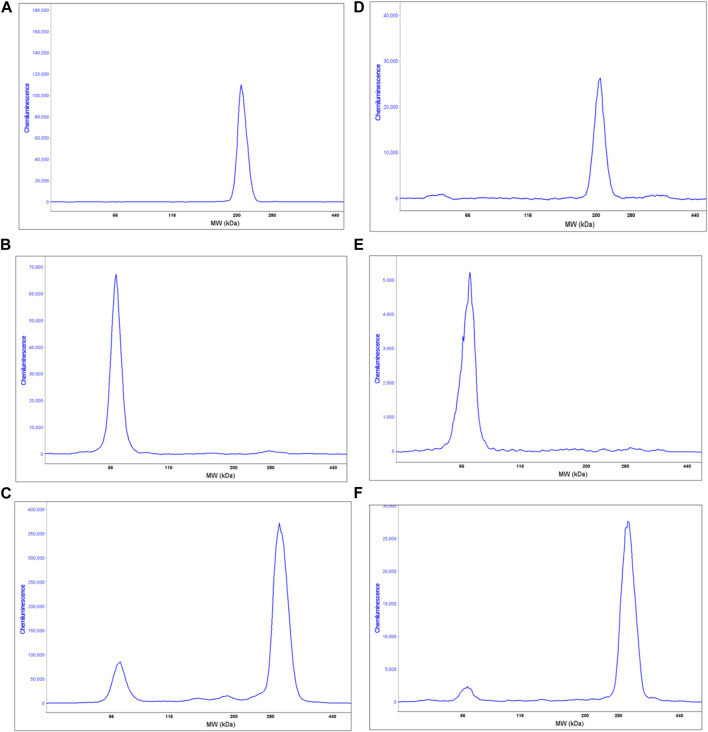
Wes analysis of proteins extracted from 1 human healthy donor non-infected and differentiated MyoD lentivirally-transfected fibroblasts and USCs. Graphs **(A, B)** show the sarcomeric myosin heavy chain expression in differentiated MyoD lentivirally-transfected fibroblasts and USCs respectively, using the anti Mf20 antibody. Graphs **(C, D)** show the DYS analysis on non-infected and on differentiated MyoD lentiviral transfected fibroblasts, respectively, using the Ab 154168 against the DMD C-terminal domain. The non-infected fibroblasts analysis shows only one peak at the molecular weight corresponding to the Dp71 DYS short isoform **(C)**. However, in differentiated MyoD-transduced fibroblasts **(D)** two peaks are present - one corresponding to the Dp71 DYS short isoform and the second to the full length Dp421 m DYS isoform. Graphs E and F show the DYS analysis on non-infected and differentiated MyoD lentivirally-transfected USCs, respectively. Like fibroblasts, non-infected USCs show only one peak, corresponding to the Dp71 DYS short isoform **(E)** while differentiated USCs show two peaks, corresponding to the Dp71 (lower) and the full length DYS isoform (higher) **(F)**.

To better evaluate myosin protein expression, proteins extracted from the same healthy donor cells tested for IHC were analysed using MF20 antibody (that recognises the myosin heavy chain, sarcomere (MHC), in the fully automated capillary system (Wes). In order to increase protein expression, especially DYS, both fibroblasts and USCs were grown in differentiation media for a total of 14 days. As expected, non-transfected cells, either fibroblasts or USCs, did not express myosin heavy chain (data not shown). On the contrary, differentiated MyoD lentiviral transfected fibroblasts show a unique peak of 212 kDa corresponding to the molecular weight expected for myosin heavy chain (Figure 5A). Similarly, differentiated MyoD lentiviral transfected USCs also have only one peak at the same molecular weight (Figure 5B).

### 4.4 DMD Dp427 m and Dp71 isoforms expression

This protocol was developed in order to generate DMD cellular models useful for drug testing and *DMD* transcript/dystrophin protein analysis. For this reason, we evaluated dystrophin expression, before and after the MyoD induction, by Wes on proteins extracted from fibroblasts and USCs. As expected, non-infected fibroblasts express only the short DYS isoform Dp71 and not the full-length muscle isoform Dp427 m (Figure 5C). Interestingly, differentiated MyoD lentivirally-transfected fibroblasts express Dp427 m but still maintain Dp71 expression (Figure 5D), unlike mature muscle fibers, that express Dp71 at very low level and only in some subjects (Kawaguchi et al., 2018) (Chesshyre et al., 2022). Similarly, non-transfected USCs expressed only the Dp71 DYS isoform (Figure 5E). After differentiation, the Dp71 isoform continued to be expressed, but at a low level, especially if compared to the Dp427 m full -length isoform (Figure 5F). Using the same cells, we evaluated the difference between the DMD Dp427 m and the Dp71 transcript expression in transfected and non-transfected fibroblasts and USCs. The Dp427 m isoform transcript is always present, even in non-muscle (non-infected) cells but with low levels of expression; Dp427 expression is 0.5% and 1% of Dp71 expression in fibroblasts and USCs, respectively. In differentiated MyoD lentivirally-transfected fibroblasts, the Dp427 transcript increased its expression, becoming comparable to Dp71, (98.6% of the Dp71 expression). Also, in the differentiated MyoD lentiviral transfected USCs, the Dp427 transcript increased its expression becoming substantially more expressed than the Dp71 isoform (Dp71 expression is 85.7% less than Dp427 expression) (Table 3).

**TABLE 3:** DMD transcripts Dp427 and Dp71 expression by qPCR. For each isoform transcript, the corresponding Ct value obtained by qPCR and the relative ratio percentage in fibroblasts and USCs without lentivirus infections (Non Myo-D induced) and after the complete differentiation induced by MyoD-lentiviral transfection (Myo-D induced) are shown. The ratio between the two isoforms is expressed as a percentage and calculated using the following formula: 2^^-(Ct Dp427−Ct Dp71)^.

		Non infected cells		differentiated MyoD lentiviral transfected	
	DMD isoforms	Ct	ratio percentage	Ct	ratio percentage
Fibroblasts	Dp427 m	31.3	0.50%	28.63	98.60%
	Dp71	23.9		28.61	
USCs	Dp427 m	31	1.10%	26	696%
	Dp71	24.6		28.8	

## 5 Discussion

Currently, 4 antisense morpholinos for treating DMD patients have been approved by the FDA, etelpilsen (exon 51) golodirsen and viltolarsen (exon 53), and casimersen (exon 45). The skipping of these exons could be beneficial for around 30% of DMD patients but skipping of other exons will be needed to treat more DMD patients (Aartsma-Rus and Corey., 2020; Anderson, 2021). To this end, more oligonucleotides need to be developed and their uptake into relevant tissues needs to be improved (e.g., by using different chemistries like peptide-phosphorodiamidate morpholino oligonucleotide (PPMO) conjugates (Moulton and Moulton, 2010). Another important aspect to be tackled is the inter-patient variability in response to the same antisense oligonucleotide (Arechavala-Gomeza et al., 2012) (Frank et al., 2020). Other therapeutic strategies for types of mutations other than deletions (i.e., nonsense mutation and readthrough therapies) and gene therapies are under testing or development (Yao et al., 2021). The availability of good cellular models is required to test these approaches *in vitro*. Considering the young average age of DMD patients and their already compromised muscular tissue, avoiding skeletal muscle biopsies and, instead, having an easy reproducible, low invasively cellular model should be a priority. In this paper, we detailed a highly efficient transdifferentiation protocol, based on the study of Kabadi and colleagues (Kabadi et al., 2015). Specifically, we used the described protocol to convert both human dermal fibroblasts and stem cells from urine into differentiated myotubes *in vitro*, using the myogenic factor (MyoD gene) delivered by a lentiviral vector. Protein and RNA isolated from both differentiated MyoD lentiviral transfected cells were used to evaluate the cell reprogramming influence on the dystrophin isoforms expression. Moreover, fibroblasts from 25 DMD patients, differentiated using the protocol we describe here, were used to test antisense PMO for the treatment of Duchenne muscular dystrophy; results of this study are summarized in Rossi et al., 2021 (Rossi et al., 2021) and Rossi et al., 2022 (Rossi et al., 2022).The protocol was set up using a commercially available line of immortalized fibroblasts to test the best multiplicity of infection (MOI) and have the highest efficiency with the lowest possible toxicity. The concentration of puromycin required to select transfected cells and the differentiation media to achieve good cell proliferation and subsequently a good fusion index, was determined. Once the protocol was established, it was extended to fibroblasts derived from 5 healthy donors and to USCs from 2 healthy donors, to assess its reproducibility and efficacy in converting cells into the myogenic lineage. All the biological replicates, either fibroblasts or USCs, gave similar immunohistochemistry results. Muscle markers, desmin and myosin heavy chain, were expressed only in MyoD-transfected cells, with the desmin signal being stronger than myosin heavy chain, in both fibroblasts and USC-derived cells. These may be explained by the nature of these markers itself; desmin is well known to be expressed at an early stage of muscle development and precedes all other known muscle structural proteins (Capetanaki et al., 1997) (Clemen et al., 2013). A concomitant factor that might have affected myosin heavy chain expression, is the time cells are left in differentiation media. Although myotubes are evident after 3–5 days in primary differentiation medium, 9 days of culture in primary differentiation medium and 5 in secondary differentiation medium (i.e., for a total of 14 days in differentiation media), give a better myogenic differentiation. For IHC evaluation, cells were maintained for 5 days in primary medium, to reduce the risk that cells would detach from the glass substrate and to confirm the presence of myotubes after a shorter differentiation time. Wes analysis for myosin and DYS were instead performed on proteins extracted from cells that had been in the primary differentiation medium for 9 days. The MF20 peak, in both transduced cell lines, is associated with high chemiluminescence, suggesting that myosin heavy chain is abundant after transfection and its expression seems to be proportionate to their time in primary differentiation medium. The expression of dystrophin isoforms before and after MyoD transfection is very interesting. The full-length Dp427 m isoform is expressed only in muscle and glial cells (Muntoni et al., 2003), while the short isoform Dp71 was believed to have a ubiquitous expression except in skeletal muscle. However, it has been recently demonstrated that the Dp71 isoform is also expressed in healthy human skeletal muscle, even if at low levels and detected only using a high amount of muscle lysate on the Wes (Kawaguchi et al., 2018). On the other hand, several studies found a higher level of Dp71 expression in DMD patients and mdx mouse muscle than in healthy subjects, suggesting a correlation between its expression and the muscle degeneration-regeneration phenomenon observed in the disease (Chesshyre et al., 2022). Using Wes, we confirmed that the Dp427 isoform is expressed only in MyoD induced fibroblasts and USCs, but these cells also expressed the Dp71 isoform at a higher level than in healthy donor skeletal muscle. Although we did not quantify this, it is possible to appreciate the different Dp71 chemiluminescent signal from the non-transfected and the transfected cells, suggesting a reduction in Dp71 expression in the latter compared to the non-transfected cells. This suggests that although MyoD can trigger myogenic conversion, it is not able to completely switch off the proteome of the original cells. In MyoD transfected fibroblasts, for example, the Dp427 m expression is part of the myogenic proteome while the Dp71 is part of the fibroblasts’ proteome. We observed that dystrophin transcripts behave differently to the corresponding proteins. The Dp427 and Dp71 transcripts are expressed in all cells but with different levels according to the cell type: Dp427 transcript is more abundant in myogenic cells, whereas Dp71 transcript is more highly expressed in non-transfected cells. These differences are seen in both our cell models. In fibroblasts, the expression of the two DMD transcripts does not change dramatically after conversion: Dp427 expression changes from being around half of Dp71 levels in non-treated fibroblasts to become similar to each other in transfected differentiated cells. USCs have a more dramatic difference in DMD isoform expression: the Dp427 transcript is present at 1% of the Dp71 transcript expression level in non-converted cells, but after myogenic conversion Dp71 expression is reduced to 14% of the expression level of the Dp427 transcript. This transcript expression evaluation was conducted only in two healthy donors: one for fibroblasts and one for USCs. USCs, obtained from urine samples, may represent an ideal source to study DMD disease (Falzarano et al., 2016) considering the great advantage over conventional cell sources collected through invasive and time-consuming procedures (Falzarano and Ferlini, 2019). But further in-depth studies on USCs are needed either to confirm our preliminary findings on DMD transcript expression differences compare to fibroblasts and to validate them as proper *in vitro* model for drug-testing. The protocol here described could help in this aim, considering that, at the moment, only three MyoD-conversion protocols are described in the literature (Falzarano et al., 2016; Kim et al., 2016; Takizawa et al., 2019) but only one uses the efficient lentivirus as vector (Kim et al., 2016). Moreover, this direct cell reprogramming protocol, is a useful way, in terms of cost and time, to obtain muscular-specific cell types than generate them by reprogramming techniques of induced pluripotent stem (Falzarano and Ferlini, 2019).

In conclusion, being able to obtain a robust patient-derived cell model capable, to some extent, to reproduce the muscle physiological and pathological phenotypes, and avoiding invasive procedures, is a great advantage for scientists and patients, especially in childhood-onset diseases such as DMD. The MyoD gene is effective in myogenic conversion of different cell types (Weintraub et al., 1989) and being able to transfect it into cells using a highly efficient integrating virus, which has low cytotoxicity, provides, in our opinion, a clear advantage compared to other methods in use. Our MyoD lentiviral transduction protocol is able to efficiently and reproducibly convert two different types of human cells, dermal fibroblasts and USCs, into the myogenic lineage. Fibroblasts have been used to test the efficacy of antisense PMO on DMD patients pre-clinically, with reliable results (Rossi et al., 2021) (Rossi et al., 2022) and USCs would be a completely non-invasive cellular model. Future work will be required to assess whether MyoD-induced USCs are indeed a good model for *in vitro* drug testing, which would substantially reduce the need for taking muscle or skin biopsies from patients.

## Data Availability

The raw data supporting the conclusion of this article will be made available by the authors, without undue reservation.
